# Hepatitis E Virus Induces Brain Injury Probably Associated With Mitochondrial Apoptosis

**DOI:** 10.3389/fcimb.2019.00433

**Published:** 2019-12-20

**Authors:** Jijing Tian, Ruihan Shi, Peng Xiao, Tianlong Liu, Ruiping She, Qiaoxing Wu, Junqing An, Wenzhuo Hao, MajidHussain Soomro

**Affiliations:** ^1^Laboratory of Animal Pathology and Public Health, Key Laboratory of Zoonosis of Ministry of Agriculture, College of Veterinary Medicine, China Agricultural University, Beijing, China; ^2^Institute of Animal Husbandry and Veterinary Medicine, Beijing Academy of Agriculture and Forestry Sciences, Beijing, China

**Keywords:** Hepatitis E virus, mitochondrial apoptosis, neuroinflammation, mongolian gerbils, human brain microvascular endothelial cells

## Abstract

Hepatitis E virus (HEV) infection has been associated with extrahepatic manifestations, particularly neurological disorders. Although it has been reported that HEV infection induced hepatocyte apoptosis associated with mitochondria injury, activation of mitochondrial apoptotic pathway in the central nervous system during HEV infection was not clearly understood. In this study, the induction of mitochondrial apoptosis-associated proteins and pro-inflammatory cytokines were detected in HEV infected Mongolian gerbil model and primary human brain microvascular endothelial cells (HBMVECs). Mitochondrial exhibited fragments with loss of cristae and matrix in HEV infected brain tissue by transmission electron microscope (TEM). *In vitro* studies showed that expression of NADPH oxidase 4 (NOX4) was significantly increased in HEV infected HBMVECs (*p* < 0.05), while ATP5A1 was significantly decreased (*p* < 0.01). Expressions of pro-apoptotic proteins were further evaluated. Bax was significantly increased in both HEV infected brain tissues and HBMVECs (*p* < 0.01). *In vivo* studies showed that caspase-9 and caspase-3 were activated after HEV inoculation (*p* < 0.01), associated with PCNA overexpression as response to apoptosis. Cytokines were measured to evaluate tissue inflammatory levels. Results showed that the release of TNFα and IL-1β were significantly increased after HEV infection (*p* < 0.01), which might be attributed to microglia activation characterized by high level of IBA1 expression (*p* < 0.01). Taken together, these data support that HEV infection induces high levels of pro-inflammatory cytokines, associated with mitochondria-mediated apoptosis. The results provide new insight into mechanisms of extra-hepatic injury of HEV infection, especially in the central nervous system.

## Introduction

Globally, there were an estimated 20 million people infected by the Hepatitis E virus (HEV) in the year of 2018 (Montpellier et al., 2018). HEV is a small non-enveloped virus with a 7.2 kb plus-strand single-stranded RNA genome and up to 8 genotypes were reported (Wang et al., 2019). Except for humans, genotypes 3 and/or 4 were observed in several domestic animals, including swine, deer, and rabbits (Kenney and Meng, 2019). Evidence for transmission of HEV-3 and HEV-4 between humans and animals has been reviewed, including waterborne transmission by accidental fecal contamination of drinking water, zoonotic transmission such as contact with infected animals or consumption of contaminated food products, and iatrogenic transmission through infected blood and blood products (Kamar et al., 2017). Though infection with HEV usually causes an acute, self-limiting form of liver inflammation, it is worth noting that extrahepatic injury of HEV, including renal diseases, reproductive system disorders, as well as pancreatitis, and a variety of neurological disorders such as Guillain-Barré syndrome, neuralgic amyotrophy, meningoencephalitis, and peripheral neuropathy, which were reported (Pischke et al., 2017; Soomro et al., 2017; Kenney and Meng, 2019; Tian et al., 2019). Since HEV infection is commonly asymptomatic or unrecognized, HEV associated extrahepatic manifestations are frequently unscreened in clinical diagnosis. Thus, it is important to understand the mechanism underlying HEV infection, especially neurological injury during infection. Mitochondria-mediated apoptosis was involved in many viral diseases. It has been reported that the release of cytochrome c was increased in BHK-21 cells infected with bovine ephemeral fever virus (BEFV) and activation of caspase-9, as well as the downstream effector caspase-3, were further detected (Lin et al., 2009). In gerbil model of HEV infection, mitochondrial damages such as mitochondria swelling, devoid of mitochondrial cristae were observed in hepatocytes via transmission electron microscope (TEM) (Yang et al., 2018). In Yang's study, up-regulation of mitochondria-mediated apoptosis regulating proteins, Bax and Bcl-2, in HEV-infected gerbils, as well as induction of activated caspase-9 and caspase-3 were observed.

Mitochondria dysfunction was reported in lots of central nervous system diseases. It has been reported that mitochondrial production of reactive oxygen species (ROS) was increased in Parkinson's disease (PD), and pro-apoptotic pathways were also activated with following activation of caspase-9, caspase-3 and Bax, which were crucial factors in mitochondria-mediated apoptosis pathway (Bose and Beal, 2016). Venezuelan equine encephalitis virus (VEEV) infection in murine brain microglial cells showed that VEEV inoculation increased cellular apoptosis, with accumulation of ROS and a marked increase of inflammatory cytokines, such as IFN-γ, IL-1α, and IL-1β (Keck et al., 2018). Thus, mitochondria play critical roles during the process of central nervous system injuries.

Previously, it was demonstrated that dysfunction of the blood-brain barrier (BBB) especially tight junction proteins contributed to the brain injury in HEV infection. However, in HEV induced brain injury, whether mitochondria-mediated apoptosis was activated or not has not been elucidated. In this study, morphological changes of mitochondria in HEV-infected gerbil brain tissues were observed by TEM. We further detected the expression of proteins that involved in mitochondria injury and significant alteration of NOX4, ATP5A1, and Bax were observed, indicating that HEV infection might facilitate pro-apoptotic signaling activation in brain tissues. Thus, caspase-9 and caspase-3 which were key effectors of mitochondrial apoptosis were examined. Both caspase-9 and caspase-3 induction were observed in cells and brain tissues that were infected with HEV. We have reported that HEV infection triggered the proliferation of microglial cells in gerbils and rabbits infected with HEV (Shi et al., 2016; Tian et al., 2019). IBA, a marker of microglial cells, was highly expressed in HEV infected brain tissues, which indicated HEV infection-induced inflammation of the brain tissues. Our study further confirmed that inflammatory cytokines such as TNFα and IL-1β were significantly induced in HEV infected brain tissues. Hence, we hypothesized that HEV induced brain tissue inflammation further promoted progress of mitochondria injury and mitochondria-mediated apoptosis. These findings provide new insights into further understanding mechanisms of extra-hepatic injury of HEV, especially the central nervous system.

## Materials and Methods

### Virus, Inoculation, and Animals

The HEV strain (CHN-HB-HD-L2, GenBank accession number KM024042) was derived from a swine liver. Shortly, the liver tissue was homogenized in 0.9% NaCl and spin down (12,000 rpm, 4°C) for 20 min. Then the supernatant was collected and filtered by a 0.22 um filter and the HEV load was tested by qPCR with a titer of 6.57 × 10^5^ genome equivalents (GE) per uL. The viral stock was stored at −86°C prior to inoculation.

72 SPF (specific-pathogen-free) male Mongolian gerbils, purchased from the Department of Experimental Animal Sciences of Capital Medical University (Beijing, China), with body weights about 50–60g were randomly assigned to 2 groups (mock group and HEV infectious group) and accommodated in separate isolators in an air-conditioned room maintained at 21–24°C with a cycle of 12 h of light and 12 h of dark for 1 week preceding the *in vivo* experiment, and feed and water were provided *ad libitum*. Gerbils in the infected group were intraperitoneally inoculated with 0.1 mL (6.57 × 10^7^ GE) of prepared viral homogenate, while gerbils in the mock group were injected with the same dose of HEV-negative swine liver homogenates.

### Sampling

Gerbils were sacrificed at 0, 7, 14, 21, 28, 42, and 56 day postinoculation (dpi). Brain and spinal cord samples were collected rapidly after euthanasia and stored at −86°C or fixed in neutral 4% paraformaldehyde or 2.5% (v/v) glutaraldehyde-polyoxymethylene solution for further study.

The levels of serum alanine aminotransferase (ALT), aspartate aminotransferase (AST), total bilirubin (T-BIL) (Yang et al., 2015) and pathological changes of the central nervous system (CNS) induced by HEV infection were examined previously (Shi et al., 2016). Positive and negative stand of HEV RNA in CNS have been tested by PCR as shown before (Shi et al., 2016). Tissues used in the present study derived from the same animals in previous research (Yang et al., 2015; Shi et al., 2016). HEV RNA-positive brain samples accompanying significant changes on liver function index collected on 14, 21, and 28 dpi were chosen for further study.

All animal protocols were approved by the Animal Care and Use Committee of China Agricultural University and were performed humanely for the alleviation of suffering.

### TUNEL Assay

TUNEL staining for apoptotic cells in the histological sections was performed using *in situ* BrdU-Red DNA fragmentation (TUNEL) Assay Kit (ab66110, Abcam) and slides were counterstained with DAPI. The images were analyzed with fluorescent microscopy. Quantification of positive signals was performed by examining number of positive cells in five randomly selected fields of each brain section.

### Immunohistochemistry Assays

The brain tissues were dehydrated and embedded in paraffin wax, and serial paraffin sections (4 μm) were obtained for immunohistochemical analysis. Immunohistochemical staining was performed using a commercial kit, according to the manufacturer's instructions (ZSGB-BIO, Beijing, China). Primary antibodies used in this study were anti-Bax (1:500, BA0315), anti-Bcl-2 (1:200, BA0412) and anti-active caspase-3 (1:300, BA3968), anti-active caspase-9 (1:300, BA0690), anti-TNF-α (1:300, BA0130), obtained from Boster, Co., Ltd., Beijing, China. Anti-IBA1 (1:500, PB0517), anti-PCNA (1:300, BS-2007R), anti-VIP (1:300, BS0077R), anti-IL-1β (1:300, BS20449R), and anti-Substance P (1:500, BS0065R) were purchased from Bioss, Co., Ltd., Beijing, China. Shortly, the sections were immersed in three consecutive 5-min xylene washes to remove paraffin and were subsequently hydrated with five consecutive ethanol washes in descending order of concentration: 100, 95, 80, 70%, and deionized water. Then, the sections were incubated with primary antibodies, respectively. The sections were executed in a moist chamber, and this procedure has been exclusively described by Ding et al. (Ding et al., 2011). The slides were finally visualized using a light microscope (LM, BX51, Olympus Co., Japan). The positive signal with brown or yellow granular mass was finally measured via the Motic Med 6.0 CMIAS Image Analysis System (Motic China Group Co., Ltd., China). The area density that represented the positive staining intensity was calculated as the ratio between the stained area and the total analyzed field. For quantitation of PCNA positive signal, positive cells in each high power field were counted. At least 5 high power fields in each slide were used for the semi-quantitative test.

### Transmission Electron Microscope (TEM)

For transmission electron microscopy, the brain samples were cut into pieces (1 × 1 × 1 mm) and fixed in 2.5% (v/v) glutaraldehyde-polyoxymethylene solution overnight at 4°C. The tissues were then washed and postfixed in 2% osmium tetroxide for 1 h at 4°C and embedded in Araldite CY212 after dehydration in ascending grades of ethanol. Ultrathin sections (50–60 nm) were cut and stained with alkaline and lead citrate uranyl acetate. The sections were examined under a JEM 1230 transmission electron microscope (JEOL, Ltd, Japan).

### Cell Culture

Primary human brain microvascular endothelial cells (HBMVECs) (BK-PM-010) were purchased from a company (Biopike Technology Company Ltd., Beijing, China) and cultured as described previously (Renou et al., 2014). Shortly, cells were inoculated with HEV (300 multiplicity of infection) and collected in 48 h for western blotting. HEV-negative homogenate served as control.

### Western Blotting

The protein concentration from HEV RNA inoculated cells and controls were determined with the BCA protein assay kit (Thermo Fisher Scientific, Waltham, MA, USA) and 20 μg of protein was separated by SDS-PAGE. The blots were incubated with anti-NOX4 (1:600), ATP5A1 (1:500), Bax (1:800), Bcl-2 (1:800), Caspase-9 (1:500), Caspase-3 (1:500) were purchased from Boster Biological Technology Co., Ltd. (China), Beijing Bioss Biological Technology Co., Ltd. (China) or ABclonal Biotechnology Co., Ltd (US), and β-actin (1:2000) purchased from Cell Signaling Technology (CST) (US), served as internal control, for 16 h at 4°C. The membranes were then incubated with the secondary antibodies conjugated with horseradish peroxidase (Santa Cruz Biotechnology) and developed by enhanced chemiluminescence (Thermo Scientific). The images were analyzed with ImageJ (NIH, Bethesda, MD, USA) Gray value was analyzed with ImageJ (NIH, Bethesda, MD, USA) to quantitatively analyze the expression levels of targeted proteins according to the level of exposure gray (resolution). Data were finally normalized to the expression of anti-β-actin.

### Quantitative and Statistical Analysis

The data represents 3 experiments analyses by one-way analysis of variance (ANOVA) or Dunnett's test using GraphPad Prism 5 (GraphPad Software). The results are expressed as the mean ± SEM. Differences are considered to be statistically significant, with ^*^p < 0.05 and ^**^p < 0.01 compared with the mock group.

## Results

### HEV Infection Induced Mitochondrial Dysfunction

**Figure 1:**
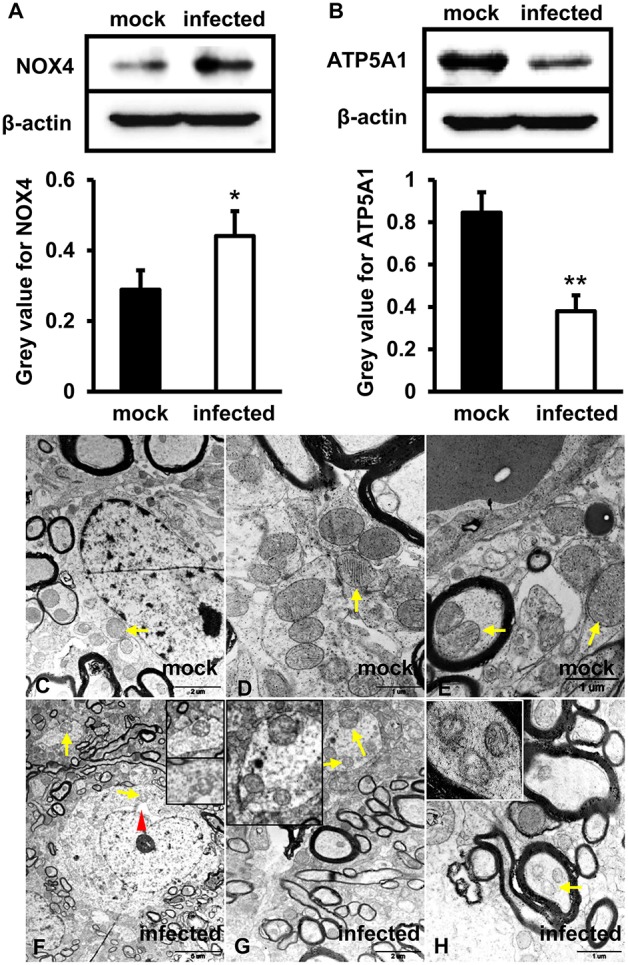
Mitochondrial dysfunction was related to an increased level of NOX4 and decreased expression of ATP5A1 in HEV infected brain tissues. **(A,B)** *in vitro*, HBMVECs were inoculated with 300 MOI HEV for 48 h for western blot, and HEV-negative homogenate served as control. Data showed that expression of NOX4 in HBMVECs treated with HEV for 48 h was significantly increased compared with mock group (^*^p < 0.05). Meanwhile, ATP5A1 was detected significantly attenuated in HEV inoculated cells (^**^p < 0.01). **(C-H)** *In vivo*, brain and spinal cord tissues that detected for HEV-RNA positive were selected for ultrastructural study. **(C–E)** Mitochondria were observed with clear cristae folded by the inner membrane in brain tissue of mock group. **(F–H)** Mitochondria with loss of cristae were found in tissues of HEV infected animals (arrows). The rough endoplasmic reticulum of neuron cells was also observed with mild distended cisternal space in HEV infected tissues (arrowhead). For western blot, gray value was analyzed with ImageJ to quantitatively analyze the expression levels of targeted proteins according to the level of exposure gray (resolution). Data were finally normalized to the expression of anti-β-actin.

To investigate the function of mitochondria in the process of HEV infection, primary human brain microvascular endothelial cells (HBMVECs) were infected with HEV. After 48 h inoculation, high levels of NOX4 were detected in HEV infected cells (*p* < 0.05) (Figure 1A), while ATP5A1 was significantly decreased in HEV infected cells (*p* < 0.01) (Figure 1B). To further detect effects of HEV infection on mitochondria morphological changes, HEV infected brain and spinal cord tissues were examined under transmission electron microscope (TEM) and fragmented mitochondria with loss of cristae and matrix in HEV infected gerbil brain tissues was observed (Figures 1F–H). Occasionally, after HEV infection, mild distended cisternal space of the rough endoplasmic reticulum was presented in neuron cells (Figure 1F). In normal brain tissue, mitochondria with intact membrane and visible cristae were showed (Figures 1C–E).

### Increased TUNEL-Positive Signals Were Detected in HEV Infected Gerbils Brain Tissue

**Figure 2:**
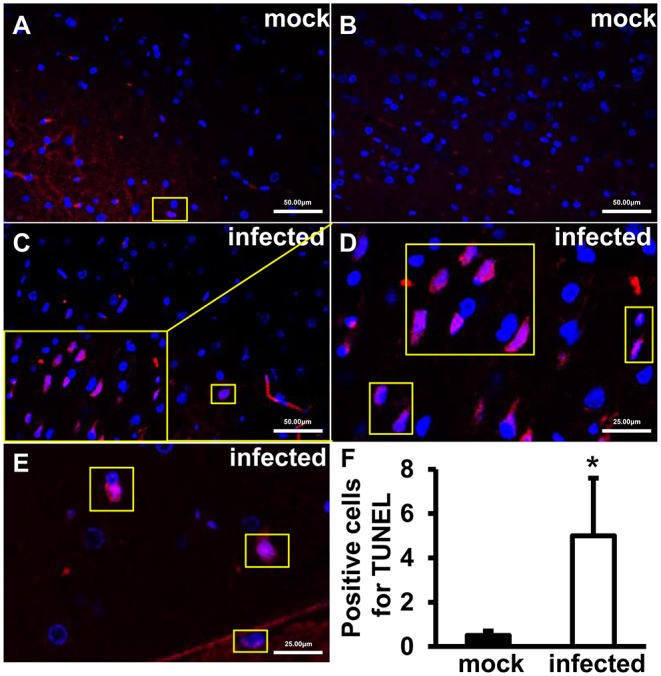
TUNEL-positive cells increased after HEV infection. TUNEL staining of the HEV-RNA positive brain tissues collected on 14, 21, and 28 dpi was performed using *in situ* BrdU-Red DNA Fragmentation (TUNEL) Assay Kit and slides were counterstained with DAPI (Blue). TUNEL-positive signals merged with DAPI were shown as purple. **(A,B)** Few positive signals were detected in animals of mock group. **(C–E)** Positive signals were exhibited in infected brain sections. The high magnification images **(D,E)** depicted granular masses of positive signals at nucleus of the apoptotic cells. **(F)** TUNEL-positive cells were significantly increased in HEV-infected gerbil brain tissues (*p < 0.05).

To explore roles of HEV infection on cell apoptosis, apoptotic cells were detected using TUNEL Assay Kit. The positive signals were mainly located in nucleus of cells in the brain. Few positive cells were detected in uninfected tissues (Figures 2A,B). More positive signals were observed in infected animals and high magnification images depicted granular masses of positive signals at nucleus of the apoptotic cells (Figures 2C–E). Further analysis showed that the apoptotic cells were significantly increased in HEV-infected brain tissues compared with uninfected animals (*p* < 0.05) (Figure 2F).

### Bax and Bcl-2 Expression Levels Increased in HEV Infected Gerbils Brain Tissue

**Figure 3:**
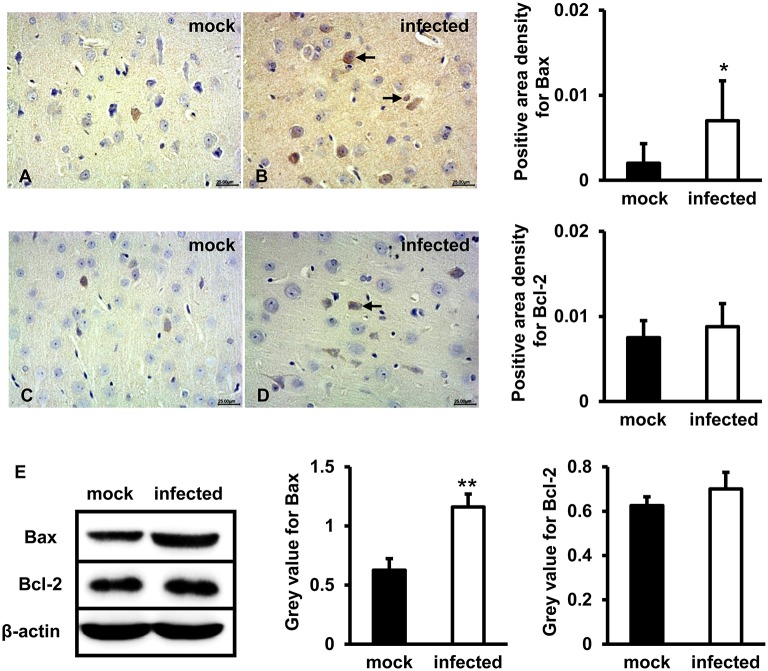
Pro-apoptotic protein Bax but not Bcl-2 was upregulated following HEV infection. **(A–D)** HEV-RNA positive brain tissues collected on 14, 21, and 28 dpi were used for the immunohistochemistry study of Bax (rabbit polyclonal IgG) and Bcl-2(rabbit polyclonal IgG). Goat anti-rabbit IgG was chosen as secondary antibody. The positive signal was measured via the Motic Med 6.0 CMIAS Image Analysis System. Data showed that Bax was mainly distributed in cytosol of neuron cells, vascular endothelial cells and few microglial cells of HEV infection tissues with increased amount compared with mock group (*p < 0.05). Bcl-2 was detected in few neurons and vascular endothelial cells in both groups. **(E)** For western blot, HBMVECs were inoculated with 300 MOI HEV for 48 h and HEV-negative homogenate served as control. Data showed that expression level of Bax was significantly higher in HBMVECs inoculated with HEV (**p < 0.01), but induction of Bcl-2 was not significant. For western blot, gray value was analyzed with ImageJ to quantitatively analyze the expression levels of targeted proteins according to the level of exposure gray (resolution). Data were finally normalized to the expression of anti-β-actin.

To test whether mitochondria-mediated apoptosis was activated by HEV challenge in brain tissues, Bax and Bcl-2 were detected. IHC study showed that positive signal of Bax protein in the brain tissue of HEV infected gerbils was mainly distributed in neurons and a small number of glial cells, and significantly upregulated following HEV infection (Figure 3B), compared with mock group (Figure 3A) (*p* < 0.05). Bcl-2 was expressed in few neurons in both HEV infected gerbils and uninfected animals, and expression level was not significantly changed (Figures 3C,D). Western blot also showed that expression of Bcl-2 was not significantly induced after HEV infection, while Bax protein was significantly increased in HBMVECs infected with HEV (*p* < 0.01) (Figure 3E).

### Caspase-9, Caspase-3, and PCNA Expression Levels Significantly Increased in HEV Infected Gerbils Brain Tissue

**Figure 4:**
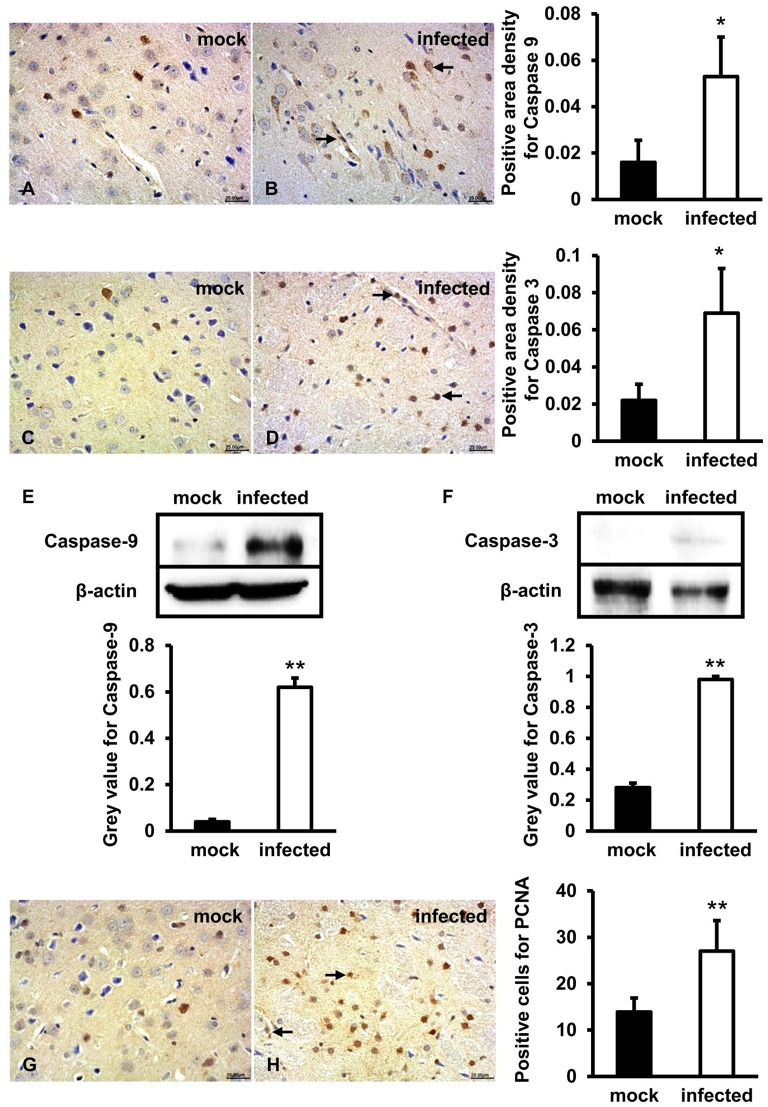
Mitochondrial apoptotic signaling was activated during HEV infection. HEV-RNA positive brain tissues collected on 14, 21, and 28 dpi were used for the immunohistochemistry study of caspase-9 (rabbit polyclonal IgG), caspase-3 (rabbit polyclonal IgG) and PCNA (rabbit polyclonal IgG). Goat anti-rabbit IgG was chosen as secondary antibody. The positive signal was measured via the Motic Med 6.0 CMIAS Image Analysis System. **(A–D)** Immunohistochemistry study showed that expression levels of activated caspase-9 and caspase-3 were expressed in neuronal cells and vascular endothelial cells of the brain tissue, with higher amount in HEV infected animals compared with mock animals (*p < 0.05). **(E,F)** For western blot, HBMVECs were inoculated with 300 MOI HEV for 48 h and HEV-negative homogenate served as control. Data showed that expression levels of cleaved caspase-9 and caspase-3 were significantly increased in HBMVECs infected with HEV compared with mock group (**p < 0.01). **(G,H)** Positive signal of PCNA was detected in glial cells and vascular endothelial cells, with significantly higher level in HEV infected brain sections compared with mock group (**p < 0.01). For western blot, gray value was analyzed with ImageJ to quantitatively analyze the expression levels of targeted proteins according to the level of exposure gray (resolution). Data were finally normalized to the expression of anti-β-actin.

To further figure out whether caspase-9 and caspase-3 were involved in this process, protein location, and expression levels were examined by both IHC and western blot. IHC showed that positive signals of activated caspase-9 and caspase-3 were distributed in few neurons and vascular endothelial cells in brain tissue of mock animals (Figures 4A,C) and more positive cells were detected in HEV infected tissue (Figures 4B,D). Western blot showed that high levels of activated caspase-9 and caspase-3 in HBMVECs infected with HEV (*p* < 0.01) (Figures 4E,F). In HEV infected animal brain tissues, PCNA positive cells were mainly distributed in glial cells and vascular endothelial cells and were significantly increased (*p* < 0.01) compared with normal brain tissues (Figures 4G,H).

### SP and VIP Expression Induced in HEV Infected Gerbils Brain Tissue

**Figure 5:**
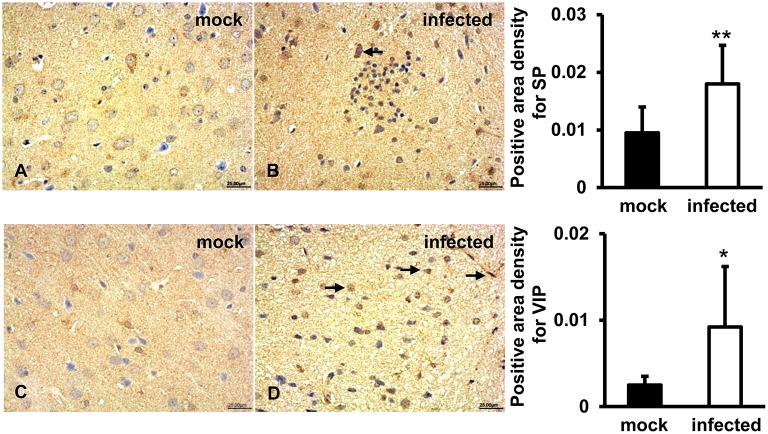
Immunoregulation of the brain tissue during HEV infection. HEV-RNA positive brain tissues collected on 14, 21, and 28 dpi were used for immunohistochemistry study of SP (rabbit polyclonal IgG) and VIP (rabbit polyclonal IgG). Goat anti-rabbit IgG was chosen as secondary antibody. The positive signal was measured via the Motic Med 6.0 CMIAS Image Analysis System. **(A,B)** A positive signal of SP was found in the cytoplasm of neuronal cells of brain tissue in both groups and was significantly induced in HEV infected animals compared with animals from mock group (**p < 0.01). **(C,D)** Expression of VIP was observed in vascular endothelial cells, astrocytes and oligodendrocytes in both groups, with significantly increased levels in HEV infected tissues compared with tissues from mock group (*p < 0.05).

To detect immunoregulatory response to HEV infection in brain tissues, SP and VIP were examined by IHC. The results showed that a positive signal of SP was distributed in the cytoplasm of some large oval and conical neurons of the gray matter of the brain in both uninfected and infected animals (Figures 5A,B). Expression of VIP was observed in vascular endothelial cells, astrocytes, and oligodendrocytes in white matter of the brain tissue in both groups (Figures 5C,D). Quantitative analysis of positive areas density of SP and VIP showed that expression levels of both proteins were significantly increased in HEV infected animals (*p* < 0.01, *p* < 0.05) (Figure 5).

### TNFα, IL-1β, IBA1 Expression Levels Induced in HEV Infected Gerbil Brain Tissues

**Figure 6:**
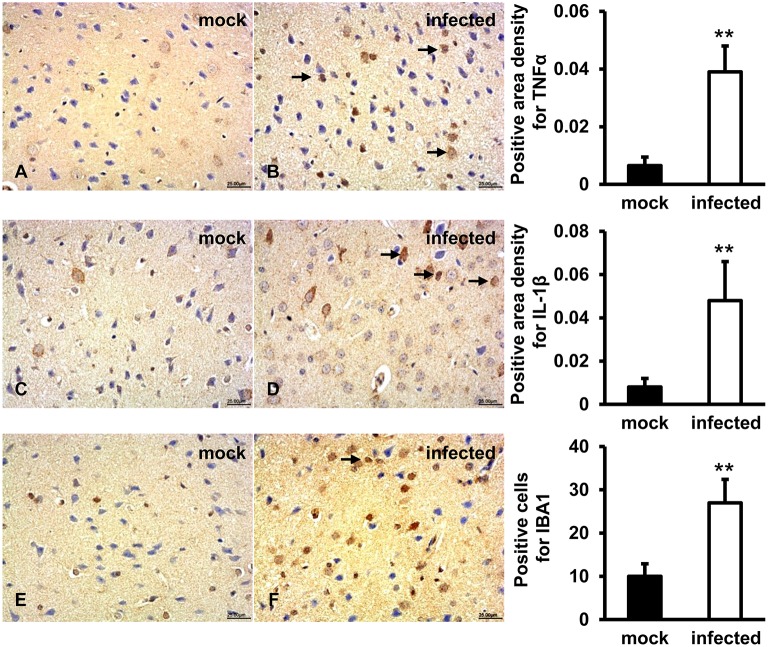
Increased pro-inflammatory responses were examined in HEV infected animal brain tissues. HEV-RNA positive brain tissues collected on 14, 21, and 28 dpi were used for immunohistochemistry study of TNFα (rabbit polyclonal IgG), IL-1β (rabbit polyclonal IgG), and IBA1 (rabbit polyclonal IgG). Goat anti-rabbit IgG was chosen as secondary antibody. The positive signal was measured via the Motic Med 6.0 CMIAS Image Analysis System. **(A,B)** Positive signal of TNFα was diffusely observed in a large number of microglial cells, astrocytes and few pyramidal neurons in HEV infected animal brain tissues, and expression level was significantly induced compared with mock group (**p < 0.01). **(C,D)** The expression level of activated IL-1β was found in microglia, astrocytes, and few neuronal cytoplasms and was significantly increased in HEV infected brain sections compared with tissues from mock group (**p < 0.01). **(E,F)** Positive expression of IBA1 was detected in microglial cells with significantly elevated levels in brain tissues infected with HEV compared with mock group (**p < 0.01).

In order to evaluate the activation of microglial cells and associated inflammatory response, TNFα, IL-1β, and IBA1 were detected by IHC. The results showed that a positive signal of TNFα was observed in few microglia and astrocytes in gerbil brain of the mock group (Figure 6A). In HEV infected animals, TNFα was diffusely distributed in a large number of microglia and astrocytes, as well as a few pyramidal neurons (Figure 6B). Expression of secretory IL-1β protein was detected in microglia, astrocytes and few neuronal cytoplasms in gerbil brain of the mock group (Figure 6C), while in HEV infected gerbils it was diffusely distributed in a large amount of microglia, astrocytes and neuron cells (Figure 6D). A positive signal of IBA1 was observed in microglial cells in brain tissue of both groups (Figures 6E,F). Quantitative analysis of positive areas density or number of positive cells of TNFα, IL1β, and IBA1 showed that expression levels of all proteins were significantly increased in HEV infected animals (*p* < 0.01) (Figure 6).

## Discussion

Our previous study showed that HEV infection could cause mitochondria damages, especially in the endothelial cells of the brain tissue. Moreover, HEV infection in the brain tissues disrupted the blood-brain barrier (BBB), especially the tight junction protein expression in endothelial cells (Shi et al., 2016; Tian et al., 2019). In the present study, primary human brain microvascular endothelial cells (HBMVCs) were chosen to further investigate the roles of mitochondria-mediated apoptotic signaling during HEV infection.

Mitochondria play a pivotal role in cellular energy-generating processes and are sensitive to cellular stress, which might further initiate programmed cell death. Both NOX4 and ATP5A1 are critical factors involved in mitochondria function and alteration of their expression levels reflected changes of mitochondria function. It has been documented that a large number of reactive oxygen species (ROS) produced by overexpression of NOX4, leading to oxidative stress in brain tissue, with further increasing the permeability of BBB and inducing neuronal apoptosis (Bedard and Krause, 2007). In our experiment, the expression of NOX4 in HBMVCs inoculated with HEV was higher than that in mock groups by western-blot assay, indicating that HEV infection up-regulated the expression of NOX4 in HBMVCs, which might lead to ROS accumulation in cells and increasing tendency to apoptosis. The study has shown that mutation of ATP5A1 gene led to fatal mitochondrial encephalopathy in newborns, which proved the importance of ATP5A1 in mitochondrial maintenance (Jonckheere et al., 2013). Our experiment demonstrated that the expression of ATP5A1 in HBMVCs infected with HEV was lower than that in the mock group by immunoblotting, indicating that HEV infection may induce mitochondrial damage and trigger ATP synthase dysfunction.

Apoptosis, a physiological process of cell death, is a form of programmed cell death (PCD) that occurs in cells during every minute of life. It can be induced through the activation of the death receptor or the mitochondrial apoptotic pathways. Mitochondria mediated apoptosis, also known as the intrinsic apoptotic pathway, is thought to be the central intracellular pathway involved in the majority of apoptosis in mammalian cells (Khosravi-Far and Esposti, 2004). TUNEL assay was known to detect apoptotic cells undergoing extensive DNA degradation (Kyrylkova et al., 2012). The morphology changes of mitochondria are critically important in apoptosis (Burke, 2017). Many death signals converge on mitochondria to activate Bak and Bax, which results in the permeabilization of the outer mitochondrial membrane and the release of pro-apoptotic factors into the cytosol and further activates caspase-9 and the apoptotic effector caspases (Alirol and Martinou, 2006; Lopez and Tait, 2015). The steady state of pro-survival Bcl-2 members could also be destroyed after the activation of Bak and Bax (McArthur et al., 2018). The previous study has shown that HEV infection led to mitochondria-mediated apoptosis of hepatocytes, such as induction of Bax, Bcl-2, caspase-9, and caspase-3 (Yang et al., 2018). In our study, increased TUNEL-positive signals were observed in infected brain tissues. Further study showed that mitochondria-mediated apoptosis was induced by HEV infection in brain tissues, including induction of pro-apoptotic protein Bax and cleaved caspase-9 and caspase-3. But expression of Bcl-2 was not significantly changed in brain tissues infected with HEV. It might be a possible reason that BBB acts effectively to protect the brain from HEV infection, while hepatic blood flow directly comes from other organs. In this condition, hepatocytes are exposed to HEV that trigger hepatocytes injury. Additionally, we observed overexpression of PCNA in brain tissue of infected HEV gerbils. It is known that neuronal cells have a high degree of differentiation and weak proliferative capacity and overexpression of PCNA in cells with weak proliferative capacity is a manifestation of DNA repair process, indicating that PCNA expression in HEV-infected brain tissues may be involved in DNA repair in responding to apoptosis. Together, HEV infection caused mitochondrial dysfunction and activation of mitochondrial apoptotic pathway. Previously, it has been reported that apoptosis involved in neuronal cell death in many of the neurological disorders, such as Alzheimer's disease and Parkinson's disease (Honig and Rosenberg, 2000). In our study, mitochondrial apoptosis might contribute to HEV induced brain injury, which might be correlated with HEV induced neurological disorders.

It has been reported that expression of substance P (SP) and vasoactive intestinal peptide (VIP) was associated with tissue injury or tissue repair, especially in inflammatory responses (Delgado et al., 2001; O'Connor et al., 2004). SP is widely distributed in the nervous system where it exerts its biological and immunological activity via high-affinity neurokinin 1 receptor (Suvas, 2017). It was shown that increased levels of SP were detected in the serum of HIV infected patients and simian immunodeficiency virus-infected rhesus macaques, and viral levels were correlated with the amount of SP released by immune cells (Johnson et al., 2016). VIP was reported to have protective effects on the focal ischemia of the brain and be a potential therapeutics for experimental autoimmune encephalomyelitis (EAE) (Deng and Jin, 2017). In our study, both induced high levels of SP and VIP were observed in HEV inoculated animals, revealing the critical immunoregulatory function of SP and VIP in HEV induced brain injury.

It has been documented that mitochondrial dysfunctions can result from abnormalities in immuno-inflammatory pathways involving elevated pro-inflammatory cytokines (Morris and Maes, 2014). TNFα was considered to play a role in hepatocytes apoptosis and liver injury and further triggered Bax activation and cleavage of caspase-9 and caspase-3 in acute liver failure (ALF) (Xu et al., 2018). Maturation of IL-1β facilitated a decrease of mitochondrial oxygen consumption, loss of mitochondrial membrane potential, depletion of ATP synthesis (Morris and Maes, 2014). Additionally, TNFα and IL-1β were reported to disturb mitochondrial function in human chondrocytes by inducing mitochondrial DNA damage, decreasing energy production and mitochondrial transcription, which correlated with the induction of apoptosis (Kim et al., 2010). In our study, increased levels of TNFα and IL-1β were detected in HEV infected brain tissues. In responding to internal or external stimuli, TNFα and IL-1β could be produced by microglia of the central nervous system. The previous study has shown that Japanese encephalitis virus infection of the central nervous tissue caused the proliferation of microglia and astrocytes, which further triggered neuroinflammatory responses (Chen et al., 2011). This work also showed that expression level of ionized calcium-binding adapter molecule 1 (IBA1), which was an activated microglial marker, was significantly increased in HEV infected animal brain sections. Combined with our previous data (Shi et al., 2016), the present results indicated that induction of the pro-inflammatory cytokines might result from proliferation of microglial cells in infected gerbils and activation and proliferation of microglial cells further contribute to damage of brain tissue in infected gerbils.

**Figure 7:**
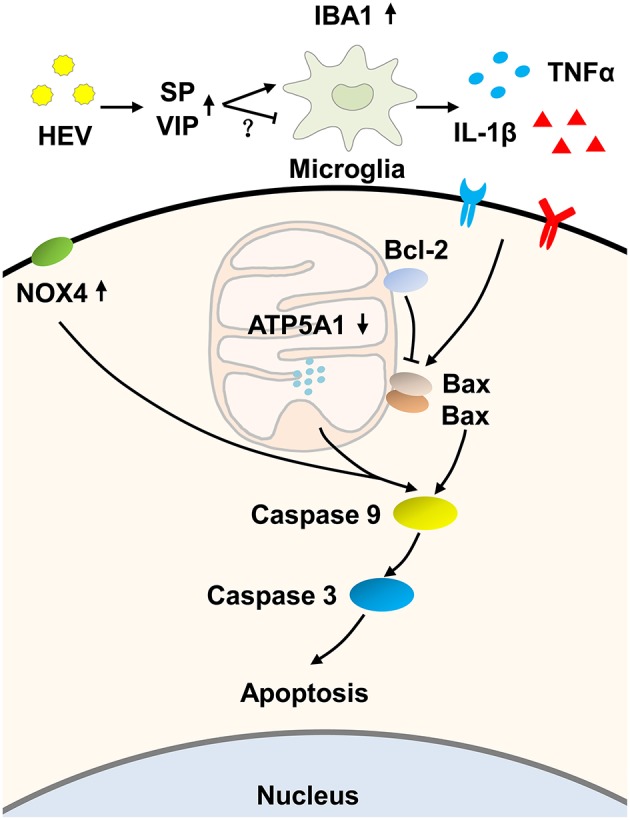
Hypothesized pathway of mitochondrial apoptosis activation induced by HEV infection. HEV entry to the brain tissue promoted the release of Substance P (SP) to activate microglial cells, while increased production of vasoactive intestinal peptide (VIP) negatively regulated activation of microglial cells as a compensatory response. Activated microglial cells produced more inflammatory cytokines such as TNFα and IL-1β. The release of TNFα and IL-1β further initiated mitochondrial apoptotic pathway. During the process, pro-apoptotic protein Bax induced ruptures of the outer membranes altered mitochondrial membrane permeability and further induced activation of caspase-9. Up-regulated expression of NOX4 and down-regulated expression of ATP5A1 also contributed to activation of caspase-9 and caspase-3 cascade and ultimately induced mitochondria-mediated apoptosis, which might be a potential way to eliminate virus but on the other hand caused neurological disorders.

In conclusion, we hypothesized that HEV infection activated the mitochondrial apoptosis of the brain tissue, according to our examination including increased TUNEL-positive signals and expression levels of Bax, cleaved caspase-9 and caspase-3. The up-regulation of PCNA might be a compensatory response to repair injured tissue. Furthermore, an increased amount of pro-inflammatory cytokines was supposed to correlate with the induction of mitochondrial apoptosis during HEV infection (Figure 7). These data present perspectives on pathogenesis of HEV induced injury of the central nervous system. More attention must be paid to the intensive study of inflammatory injury and mechanisms controlling apoptosis during HEV infection.

## Data Availability Statement

All datasets generated for this study are included in the article/supplementary material.

## Ethics Statement

The animal study was reviewed and approved by Animal Care and Use Committee of China Agricultural University.

## Author Contributions

JT, RShi, and RShe performed the study concept and design. RShi, PX, TL, QW, and JA performed the laboratory work and data analysis. WH and MS performed the analysis and interpretation of data. JT and RShi wrote the paper. All of the authors read and approved the final article.

### Conflict of Interest

The authors declare that the research was conducted in the absence of any commercial or financial relationships that could be construed as a potential conflict of interest.
